# The Molecular Mechanism of Vitamin E as a Bone-Protecting Agent: A Review on Current Evidence

**DOI:** 10.3390/ijms20061453

**Published:** 2019-03-22

**Authors:** Sok Kuan Wong, Nur-Vaizura Mohamad, Nurul ‘Izzah Ibrahim, Kok-Yong Chin, Ahmad Nazrun Shuid, Soelaiman Ima-Nirwana

**Affiliations:** Department of Pharmacology, Faculty of Medicine, Universiti Kebangsaan Malaysia, Jalan Yaacob Latif, Bandar Tun Razak, Cheras, Kuala Lumpur 56000, Malaysia; jocylnwsk@gmail.com (S.K.W.); vaizuramohd@gmail.com (N.-V.M.); nurulizzah88@gmail.com (N.I.I.); chinkokyong@ppukm.ukm.edu.my (K.-Y.C.); anazrun@yahoo.com (A.N.S.)

**Keywords:** inflammation, osteoblast, osteoclast, oxidative stress, tocopherol, tocotrienol

## Abstract

Bone remodelling is a tightly-coordinated and lifelong process of replacing old damaged bone with newly-synthesized healthy bone. In the bone remodelling cycle, bone resorption is coupled with bone formation to maintain the bone volume and microarchitecture. This process is a result of communication between bone cells (osteoclasts, osteoblasts, and osteocytes) with paracrine and endocrine regulators, such as cytokines, reactive oxygen species, growth factors, and hormones. The essential signalling pathways responsible for osteoclastic bone resorption and osteoblastic bone formation include the receptor activator of nuclear factor kappa-B (RANK)/receptor activator of nuclear factor kappa-B ligand (RANKL)/osteoprotegerin (OPG), Wnt/β-catenin, and oxidative stress signalling. The imbalance between bone formation and degradation, in favour of resorption, leads to the occurrence of osteoporosis. Intriguingly, vitamin E has been extensively reported for its anti-osteoporotic properties using various male and female animal models. Thus, understanding the underlying cellular and molecular mechanisms contributing to the skeletal action of vitamin E is vital to promote its use as a potential bone-protecting agent. This review aims to summarize the current evidence elucidating the molecular actions of vitamin E in regulating the bone remodelling cycle.

## 1. Introduction

Bone is a dynamic connective tissue that continuously undergoes formation and resorption processes throughout life. This is known as bone remodelling [1]. Bone resorption precedes bone formation in the bone remodelling cycle. The former involves the removal of mineralized bone by osteoclasts and the latter involves the formation of bone matrix by osteoblasts. The balance between bone resorption and bone formation is crucial for sustaining bone mass and maintaining systemic mineral homeostasis, thus preserving bone health. The direct interaction among osteoblasts, osteoclasts, and osteocytes, organised by various systemic and local factors, facilitates precise bone remodelling [2]. The activation of bone-forming or bone-resorbing activities is governed by the receptor activator of nuclear factor kappa-B (RANK)/receptor activator of nuclear factor kappa-B ligand (RANKL)/osteoprotegerin (OPG), canonical Wnt/β-catenin, and oxidative stress signalling pathways [2]. The uncoupling of the bone remodelling process, explained by either an increase in bone resorption or a decrease in bone formation, often results in the progression of pathological bone diseases, like osteoporosis [3]. Due to the high prevalence and growing healthcare burden of osteoporosis, much effort has been invested in the search for new anti-osteoporotic agents that can mitigate the progression of osteoporosis.

Vitamin E is an essential lipid-soluble vitamin that consists of two subgroups: tocopherol (TF) and tocotrienol (T3). Both TF and T3 have a chromanol ring with a long carbon phytyl chain, distinguishable by the presence of solely single bonds or three double bonds in the hydrocarbon tail, respectively. Each isoform can be further divided into four distinct analogues, namely alpha (α), beta (β), gamma (γ), and delta (δ), based on the location of the methyl groups in the chromanol nucleus. The effects of vitamin E on bone in animal experimentations have been extensively reviewed [4,5]. Specifically, researchers revealed heterogeneous findings on the bone-protective properties of αTF [6,7,8,9]. High levels of αTF were associated with negligible effects on bone in young adult normal mice. However, increased bone biomechanical strength was seen in old normal mice [10]. Meanwhile, preclinical studies consistently showed that palm T3 (containing a mixture of TF and T3 isomers), annatto T3 (containing a mixture of T3 isomers), and individual T3 isomers exhibited potential skeletal-promoting effects in healthy animals [11,12] as well as in osteoporotic animals induced by various stressors, such as ovariectomy [13,14,15,16,17], orchidectomy [18,19,20,21], nicotine [22,23,24], alcohol [25], free radicals [26], glucocorticoid [27,28], buserelin [29,30], and metabolic syndrome (MetS) [31,32,33]. In comparison, between the two subgroups of vitamin E, some studies also reported that αTF exhibited comparable [34,35] or less beneficial effects than T3 on the skeletal system [11,22,25]. Since the bone-sparing effects of vitamin E in vivo are quite established, the molecular mechanisms of vitamin E in achieving these actions are of major interest to scientists.

This review highlights the recent advances in our understanding of the molecular aspects of vitamin E as a bone-protecting agent, particularly through crosstalk between various signalling pathways and signal transduction molecules. We hope to provide the readers with current updates on the molecular machinery of vitamin E in promoting bone health and the research gap to be bridged by investigators in order to promote vitamin E as an alternative therapeutic agent for osteoporosis.

## 2. The Effects of Vitamin E on Bone Cells

The direct effects of vitamin E on bone cells have been previously investigated. In an earlier study, Soeta et al. (2010) explored the effects of vitamin E (αTF and δTF) on osteoblast differentiation. It was shown that TF inhibited osteoblast differentiation at the early stage, indicated by decreased alkaline phosphatase (ALP) activity and expression of osteocalcin (OCN). Osteoblast differentiation returned to normal at a later stage, whereby ALP activity and expression of OCN were comparable between the TF-treated and normal control groups [36]. Another study by Ahn et al. (2011) found that proliferation of human mesenchymal stem cells (MSCs) was enhanced after incubation with αTF. The expression of ALP and runt-related transcription factor-2 (Runx-2) was significantly increased in the αTF-treated cells [37]. On the other hand, Urban et al. (2012) reported that αTF could not improve proliferation and differentiation of primary bovine osteoblasts as the elevation of collagen I, osteonectin, and OCN was not detected [38]. For osteoclasts, Kim et al. (2012) revealed that α-tocopheryl succinate reduced the expression of RANKL in osteoblasts as well as inhibited osteoclastogenesis in co-cultures of mouse bone marrow cells and calvarial osteoblasts stimulated by interleukin-1 (IL-1) [39]. In line with previous findings, an ex vivo experiment performed by Johnson et al. (2016) also showed that the osteoclast number (Oc.N), and monocyte and lymphocyte production were suppressed in ovariectomized rats fed a diet with supplemental D-α-tocopherol acetate (300 mg/kg diet) for 100 days. The results reiterated that TF suppressed osteoclast formation, possibly by inhibiting monocyte and lymphocyte production [40].

Gamma-T3 was shown to improve proliferation (as shown by increased ALP, OCN, osteonectin, and collagen I), differentiation (as shown by upregulation of Runx-2 expression), and mineralization (as shown by increased bone nodules), and reduced apoptosis (as shown by low caspase-3 activity) of osteoblastic cells [41,42]. In addition, Wan Hassan et al. (2018) demonstrated that annatto-derived T3 (consists of 10% γT3 and 90% δT3) enhanced osteogenic activity of murine osteoblasts by promoting the expression of bone formation-related genes. Annatto T3 increased the expression of osterix (OSX), collagen type 1 alpha 1 (COL1α1), ALP, and OCN, resulting in increased formation of collagen fibrils and mineralization of the extracellular matrix [43]. The effects of T3 isomers on human osteoclasts were investigated by Brooks et al. (2011). Both αT3 and γT3 isomers suppressed osteoclastogenesis, with the latter exhibiting more potent inhibition on osteoclast formation and activity than the former [44].

Comparison between TF and T3, αT3 and δT3 exhibited more superior effects in promoting osteoblast differentiation than αTF and δTF when the nano-emulsion mode of delivery was applied. Higher expression of OSX, collagen I, and ALP activity were observed in primary human MSCs after exposure of T3 relative to exposure of TF [45]. Potency between individual vitamin E isomers were compared by using bone scaffolds cultured with osteoblasts or co-cultured with osteoblasts and osteoclasts. These cultures and co-cultures were treated with 100 nM of αTF, αT3, βT3, γT3, and δT3 for 28 days. In the three-dimensional (3D) osteoblast culture model, scanning electron microscope (SEM) analysis showed attachment and clumping of osteoblasts on the surface of αT3-, γT3-, and δT3-treated bone scaffolds. Micro-computed tomography (micro-CT) analysis showed increased bone volume (BV), trabecular thickness (Tb.Th), and trabecular number (Tb.N) in bone scaffolds treated with γT3 and δT3. In the 3D osteoblast-osteoclast co-culture model, bone scaffolds incubated with γT3 had higher BV, Tb.Th, and Tb.N and lower bone porosity. A biomechanical strength test also showed an increase in elasticity in the bone scaffolds treated with all types of vitamin E isomer, particularly γT3 and δT3. This study suggested that γT3 and δT3 might be the most active isomers acting on bone [46].

Overall, the existing studies indicated that the effects of αTF were heterogeneous whereas the protective effects of T3 were consistent on osteoblastic cells. Meanwhile, the in vitro studies that examine the effects of vitamin E (TF and T3) on osteoclasts are limited. 

## 3. Molecular Actions of Vitamin E on Bone

### 3.1. The Macrophage-Colony Stimulating Factor (M-CSF) and RANK/RANKL/OPG Pathway

The M-CSF and RANK/RANKL/OPG trimolecular complex system play an important role in osteoclast formation and the regulation of bone resorption. The expression of M-CSF by osteoblastic stromal cells is required for the differentiation of osteoclast precursor cells into mature osteoclasts in the presence of RANKL [47,48,49]. RANKL is a member of the tumour necrosis factor superfamily, which is typically membrane bound on osteoblastic and activated T cells [47]. It is also commonly referred to as an OPG ligand, osteoclast differentiation factor (ODF), or tumour necrosis factor related activation-induced cytokine (TRANCE). The RANK messenger ribonucleic acid (mRNA) was found to be highly expressed in bone marrow-derived osteoclast progenitors and mature osteoclasts [50]. In vivo studies demonstrated that RANK-knockout mice experienced osteopetrosis or bone hardening. This is due to an absence of osteoclasts as a result of an inability of osteoblasts to support osteoclastogenesis [51,52]. In contrast, an activated mutation in exon 1 of RANK in humans was reported to account for the increase in osteoclast formation, activities, and osteolysis. This condition often occurs in some patients with familial Paget’s disease of the bone with an autosomal recessive disorder [53].

The binding of RANKL to its receptor, RANK, on cells of the myeloid lineage activates a cascade of intracellular signalling events, including interactions with tumour necrosis factor receptor-associated factor 6 (TRAF6) adaptor molecules, thereby stimulating the activation of nuclear factor-kappa B (NF-κB), nuclear factor of activated T-cells cytoplasmic 1 (NFATc1), mitogen-activated protein kinase (MAPK), and phosphatidylinositol-3-kinase (PI3K), responsible for osteoclast formation, activation, and survival [54,55,56]. NF-κB has been shown to mediate RANKL-induced osteoclast differentiation, leading to activation of Fos proto-oncogene (c-Fos) prior to NFATc1 [57]. Described as the master regulator of osteoclast formation, the presence NFATc1 is important in the differentiation of stem cells into osteoclasts [58]. It is activated transiently in osteoclast precursors within an hour with the presence of RANKL and NF-κB p65 interaction [59]. The proto-oncogene, c-Fos, also plays an essential role in the osteoclastic differentiation of precursors generated by M-CSF and RANKL. A study found that overexpression of c-Fos in mice lacking c-Fos macrophages resulted in the up-regulation of RANK expression through induction of the RANKL signals [59].

OPG is a protein secreted mainly by cells of the osteoblast lineage and plays a role as a natural key endogenous regulator of the RANK/RANKL/OPG pathway. Both OPG and RANK are receptors which show affinity to the same ligand RANKL [60]. Acting as a decoy receptor by binding with RANKL, OPG thereby inhibits osteoclastogenesis and the survival of pre-existing osteoclasts [48,61,62]. Generally, RANKL expression is up-regulated when OPG expression is down-regulated or not induced to the same degree as RANKL, suggesting that the ratio of RANKL/OPG represents an important determinant of bone resorption [63,64]. OPG overexpression significantly increases the cortical and cancellous bone mass in association with reduced osteoclast numbers [65]. In a study utilizing OPG-knockout mice, OPG deficiency increased cortical porosity and reduced cortical and cancellous bone volume and density [66]. Additionally, mice subjected to ovariectomy (animal model for postmenopausal osteoporosis) followed by immediate treatment with adenoviral human OPG fusion protein were found to have a higher bone volume with reduced osteoclast numbers in axial and appendicular bones after 4 weeks [67]. Another study further demonstrated that a single intravenous OPG injection in rats caused a significant increase in tibial cancellous bone volume and femoral bone mineral density (BMD) with a significant reduction in osteoclast surface [68]. A single subcutaneous injection of OPG was found to be effective in rapidly and profoundly reducing bone turnover for a sustained period in postmenopausal women. Thus, elevating the level of OPG might be effective in the treatment of bone diseases characterized by increased bone resorption, such as osteoporosis [69].

Therapeutic approaches in the treatment of pathological bone loss targeting this system have been widely investigated. A growing body of literature delineated the biological properties of the natural vitamin E and its various forms in the maintenance of bone metabolism. Interestingly, an in vitro study by Lee et al. (2009) showed that Trolox (a hydrophilic derivative of TF with a carboxylic group) inhibited osteoclast formation in bone marrow cell osteoblast co-culture by suppressing the IL-1-induced RANKL expression in a dose-dependent manner (100, 200, and 500 µM) [70]. An in vivo study also demonstrated that OPG levels were increased in nicotine-induced osteoporotic rats after three months treatment with 60 mg/kg αTF [24]. The anti-resorptive potential of αT3 was demonstrated using a co-culture of osteoblasts and bone marrow macrophages [71]. Treatment with αT3 at 50 µM was discovered to suppress RANKL expression in osteoblast, RANKL-induced expression of c-Fos, NFATc1, acute activation of extracellular signal-regulated kinase (ERK), NF-κB activation, and pit formation (indicative of osteoclast resorptive activity) by mature osteoclasts [71]. Administration of γT3 at 100 mg/kg via subcutaneous injection once a month for three months has been shown to significantly inhibit the increase of RANKL expression and block the decrease of OPG expression in female ovariectomized mice [15]. A recent study also evaluated the effects of annatto-derived T3 composed of 90% δT3 and 10% γT3 using a testosterone-deficient osteoporotic rat model through orchidectomy. The results indicated that annatto T3 at 60 mg/kg significantly decreased the osteoclast-related gene of RANKL expression when compared with the orchidectomized group [19].

Apart from that, Shen et al. (2018) investigated the potential of annatto T3 containing 90% δT3 and 10% γT3 with 70% purity in protecting bone in postmenopausal osteopenic women who are at increased fracture risk [72]. Clinical findings indicated that the level of the placebo group had the highest soluble RANKL (sRANKL) and sRANKL/OPG ratio among the placebo (430 mg olive oil/day), low T3 (430 mg T3/day), and high T3 (860 mg T3/day) groups, suggesting a higher rate of bone resorption. The changes in the RANKL/OPG ratio at 6 and 12 weeks were observed and results showed a significant reduction in both low (6.81% and 12.47%) and high levels of T3 (12.8% and 23.78%), respectively. These findings further confirmed that the beneficial properties of dietary T3 supplementation on bone were mediated through suppression of osteoclastic activity [72].

Overall, the RANK/RANKL/OPG system is an essential signalling pathway involved in bone cell to cell communication, with ample evidence showing that modification of this signalling pathway has major effects on bone remodelling. RANKL mediates osteoclastogenesis and activates mature osteoclasts, whereas OPG negatively regulates the binding of RANKL to RANK and reduces the half-life of membranous RANKL, thus inhibiting bone resorption induced by osteoclasts. Accumulated evidence has reported that treatment with T3 shows promising bone protective effects by modulating this pathway. However, further studies are needed to strengthen the findings.

### 3.2. Pro-Inflammatory Cytokines

Inflammation is the frontline defence against diseases characterised by the activation of immune cells in the innate and adaptive immune system, leading to the production of inflammatory cytokines. Inflammation plays an important role in eliminating damaged tissues and initiating tissue repair. However, the perpetuation of the inflammatory response in turn inhibits bone formation and activates bone resorption. Cytokines are the predominant players in regulating the inflammatory response observed in metabolic bone diseases, including osteoporosis. These cytokines are, but not limited to, tumour necrosis factor-alpha (TNF-α), interleukin (IL), and interferon (IFN). The relationship between vitamin E (particularly αTF), inflammation, and bone health has been investigated in a large cohort study of older women consisting of two visits (first visit: 1997–1999, age: 55 ± 2.2 years, *n* = 3883; second visit: 2007–2011, age: 66 ± 2.2 years, *n* = 2130). In this study, Yang et al. (2016) found a positive association between serum αTF and BMD at the femoral neck, but a negative association between serum αTF and inflammation, evidenced by higher concentrations of IL-6 and high-sensitivity C-reactive protein (hs-CRP), among participants not taking vitamin E supplements [73].

Pro-inflammatory mediators have been previously demonstrated to positively and negatively regulate osteoclast and osteoblast functions, respectively. TNF-α, IL-1, and IL-6 are potent bone resorption stimulators. The recognition of TNF-α and IL-6 by their respective receptors on bone marrow stromal cells results in the downregulation of osteoblast gene products, partly via the inhibition of MAPK, the activation of suppressor of mothers against decapentaplegic ubiquitylation regulatory factor 1 (SMURF1) and SMURF2, as well as the activation of signal transducers and activators of transcription (STAT) [74]. Pro-inflammatory cytokines also suppress the transcription process of osteogenic factors through upregulation of Dickkopf-related protein 1 (DKK1) and sclerostin (SOST) to inhibit the Wnt/β-catenin pathway [74]. In addition, the interaction between inflammatory cytokines and their receptors increases the production of M-CSF and RANKL by osteoblasts, thus promoting osteoclast differentiation, proliferation, and activation [75]. In osteoclasts or osteoclast precursors, TNF-α, IL-1, and IL-6 signal via the NF-κB, MAPK, and Janus kinase (JAK)-STAT pathways to upregulate osteoclast-related genes and further amplify osteoclastogenesis [74]. Both TNF-α and IL-1 also have potent anti-apoptotic effects on osteoclasts, suggesting that IL-1 works intimately with TNF-α to favour inflammatory osteoclastogenesis and bone destruction [75]. 

Other resorptive cytokines, such as IL-2 and IL-23, have been recently examined for their role in the disease state of osteoporosis. They were initially recognised as inflammatory cytokines that interconnect immune cells, osteoclasts, and synoviocytes, thus playing a crucial role in the pathogenesis of rheumatoid arthritis [76]. The role of these cytokines in osteoporosis has been of particular interest as they affect the differentiation and activation of pathogenic osteoclasts. Both IL-2 and IL-23 are known as T cell growth factor to facilitate the expansion of T cell populations [76,77]. T cells are the main source of other pro-inflammatory cytokines, such as TNF-α, IL-6, and IL-17 [75]. The interaction between IL-23 and IL-23 receptors also transduces signal to induce the secretion of TNF-α, IL-1, and IL-23 [76]. Thus, IL-2 and IL-23 contribute to the positive feedback to the inflammation cascade, subsequently promoting the persistent release of RANKL by osteoblasts.

On the other hand, IFN-γ has biphasic effects on bone metabolism both in vitro and in vivo. IFN-γ potentially promotes osteoblastogenesis through upregulation of various osteogenic factors, such as Runx-2, OSX, ALP, and OCN, but inhibits adipogenesis through downregulation of peroxisome proliferator-activated receptor-gamma (PPAR-γ) [78,79]. The underlying mechanisms of IFN-γ in reducing osteoclast formation have been postulated through the downregulation of colony-stimulating factor-1 receptor (c-Fms), NFATc1, and TRAF6, subsequently impairing the activation of downstream targets, including NF-κB and c-Jun N-terminal kinase (JNK) [80,81]. IFN-γ also stimulates osteoblasts to produce nitric oxide and enhances Fas–Fas ligand interaction to induce osteoclast precursor apoptosis [82,83]. Conversely, IFN-γ indirectly promotes osteoclast differentiation by stimulating T cell activation and T cell secretion of osteoclastogenic factors, including TNF-α and RANKL [84]. IFN-γ also induces the expression of dendritic cell-specific transmembrane protein (DC-STAMP), which is responsible for the fusion of mononucleated osteoclasts into functional mature osteoclasts [85]. Hence, IFN-γ plays conflicting roles in osteoclastogenesis by inhibiting early stages, but promoting late stages of osteoclast formation.

Monocyte chemoattractant protein-1 (MCP-1) is a chemokine that plays a role in the migration and infiltration of monocytes or macrophages in response to inflammation [86]. MCP-1 is specifically expressed in osteoclasts. A previous in vivo study reported that multinuclear osteoclast differentiation was inhibited and NFATc1 was downregulated in osteoclasts derived from MCP-1-deficient mice [87]. Furthermore, MCP-1 did not induce cell–cell fusion in osteoclasts with a DC-STAMP deficiency (DC-STAMP is a direct target of NFATc1, which is highly induced during osteoclast differentiation) [88]. These findings suggested that MCP-1 was implicated in the regulation of osteoclast differentiation and fusion through upregulation of NFATc1 and in the presence of DC-STAMP.

A wide array of laboratory studies demonstrated the osteoprotective effects of vitamin E through the reduction of systemic inflammation. Oestrogen deficiency caused by ovariectomy increased the levels of IL-1 and IL-6 in animals. Treatment with 60 mg/kg palm-derived T3 (37.2% αT3, 39.1% γT3, and 22.6% δT3) successfully prevented a rise in cytokine levels [89]. Metabolic syndrome, defined as a medical condition with cardiovascular risk factors, such as obesity, hypertension, hyperglycaemia, and hypercholesterolemia, was also associated with the occurrence of inflammation [31,32,33] and skeletal impairment [90,91]. Palm-derived T3 (21.9% αTF, 24.7% αT3, 4.5% βT3, 36.9% γT3, and 12.0% δT3) and annatto-derived T3 (16% γT3 and 84% δT3) reduced the levels of inflammatory markers (IL-1α and IL-6), concurrent with improvements of bone microstructure in the MetS animals [31,32,33]. Recently, it was also found that high-fat diet-induced type 2 diabetes caused inflammation as well as degeneration of bone microstructure in the femur and lumbar of male mice [92]. In this study, the authors found that annatto T3 (10% γT3 and 90% δT3) improved glucose and insulin tolerance, elevated serum procollagen I intact N-terminal propeptide (P1NP, a bone formation marker), decreased serum carboxyl-terminal telopeptide of type I collagen (CTX, a bone resorption marker), improved trabecular and cortical microstructure, as well as suppressed the expression of inflammation markers (MCP-1, IL-2, IL-23, IFN-γ, and TNF-α) in the liver of high-fat diet-induced diabetic mice [92].

The distinct effects of T3 and αTF on inflammatory markers were also elucidated using free radical- and nicotine-treated animals. Free radicals generated by ferric nitrilotriacetate elevated bone resorbing cytokines (IL-1 and IL-6), reduced OCN levels, and impaired bone histomorphometry (lowered osteoblast number (Ob.N), bone formation rate (BFR), BV, Tb.Th, raised eroded surface (ES), and Oc.N). All these adverse changes were reversed by supplementation of 100 mg/kg of a palm oil T3 mixture (30.7% αT3, 55.2% γT3, and 14.1% δT3). However, similar doses of αTF only reduced ferric nitrilotriacetate-induced increase of Oc.N and IL-6 in the animals, suggesting that the palm-derived T3 mixture was better than αTF [26]. Two months of nicotine treatment also increased inflammatory cytokines (IL-1 and IL-6) and bone-resorption markers (pyridinoline, PYD) while reducing a bone formation marker (OCN). Three types of vitamin E (αTF, T3-enhanced fraction, (42.98% αT3, 30.98% γT3, 13.81% δT3, 12.23% palm oil devoid of αTF), and γT3) at a dose of 60 mg/kg showed effectiveness in improving all these parameters compared to the negative control group. In addition, only T3-enhanced fraction and γT3 showed higher levels of OCN compared to the normal control animals. These results suggested that T3 was superior to αTF in reversing the adverse effects of nicotine on the skeleton [23]. In another study, Norazlina et al. (2007) revealed that 60 mg/kg of a palm T3 mixture (composition not mentioned) was able to prevent increments of IL-1 and IL-6 due to nicotine treatment. In contrast, treatment with αTF at a similar dose increased the levels of IL-1 and IL-6 in the nicotine-treated animals [93]. 

Taken together, the data from these published papers revealed the skeletal-protecting properties of vitamin E via its anti-inflammatory properties using various in vivo osteoporotic models. The anti-inflammatory activities of T3 seem to be more effective than αTF in osteoporotic conditions. Furthermore, TNF-α, IL-1, IL-2, IL-6, IL-23, and MCP-1 are well-known for their anti-osteogenic and osteoclastogenic effects. However, most of these studies mainly assessed the effects of vitamin E on the levels of IL-1 and IL-6. A paucity of data has shown the effects of vitamin E on other bone-resorbing cytokines. IFN-γ has biphasic control of bone metabolism. We believe both the positive and negative effects of IFN-γ on bone exist in parallel, but the net outcome of IFN-γ remains unresolved. Thus, the role and exact mechanism of IFN-γ requires further clarification. There are other inflammatory cytokines that have been recognised as osteoclastogenic (such as IL-7, IL-8, IL-11, IL-15, IL-17, and IL-34) and anti-osteoclastogenic cytokines (such as IFN-α, IFN-β, IL-3, IL-4, IL-10, IL-12, IL-27, and IL-33). The role of vitamin E on these cytokines in bone homeostasis awaits further investigation.

### 3.3. Reactive Oxygen Species (ROS) and the Anti-Oxidant System

Oxidative stress refers to a condition in which an imbalance occurs between ROS generated from various oxidation pathways and the antioxidant defence system in the body, leading to enhanced lipid peroxidation, protein modifications, and deoxyribonucleic acid (DNA) damage [94]. A certain physiological level of ROS is essential for cellular functions, but over-generation of ROS is associated with the pathogenesis of multiple human diseases, such as cardiovascular disease, diabetes, hypertension, cancer, and musculoskeletal disorders. In bone metabolism, oxidative stress positively correlates with osteoclast differentiation and osteoblast apoptosis, but negatively correlates with osteoblast activity and osteoclast apoptosis [95,96,97].

Mechanistically, ROS promotes osteoclastogenesis and inhibits osteoclast apoptosis via an increase of RANKL production and activation of the NF-κB-mediated inflammatory response [97]. The inhibition of osteoblastogenesis and stimulation of osteoblast apoptosis by ROS are associated with antagonisation of Wnt/β-catenin signalling through diversion of β-catenin from T cell factor-(Tcf-) to Forkhead Box O-(FoxO-)mediated transcription and the direct activation of NF-κB [98,99]. Increased lipid peroxidation also activates PPAR-γ, thus favouring adipogenesis and inhibiting osteoblastogenesis [100]. All these events contribute to increased bone turnover, reduced mineralization, and, subsequently, bone loss. The natural enzymatic and non-enzymatic anti-oxidant systems are essential in inactivating ROS and relieving oxidative stress. The enzymatic anti-oxidants include superoxide dismutase (SOD), glutathione peroxidase (GPx), and catalase (CAT) whereas the non-enzymatic antioxidants include reduced glutathione (GSH). ROS, such as superoxide radical anion (O_2_^•−^), is the by-product generated by the oxidation of nicotinamide adenine dinucleotide phosphate (NADPH) oxidase. Excess O_2_^•−^ is removed through SOD, which catalyses the disproportionation of O_2_^•−^ to the less reactive hydrogen peroxide (H_2_O_2_) and oxygen (O_2_) (Equation: 2O_2_^•^ + 2H^+^ → H_2_O_2_ + O_2_). H_2_O_2_, generated by SOD, can be further inactivated by CAT or GPx. CAT catalyses the decomposition of H_2_O_2_ to water (H_2_O) and O_2_ (Equation: 2H_2_O_2_ → 2H_2_O and O_2_) whereas GPx metabolizes H_2_O_2_ to H_2_O using GSH as a hydrogen donor, converting two GSH molecules to its oxidised form (GSSG) (Equation: H_2_O_2_ + 2GSH → 2H_2_O + GSSG) [94,101].

Since the potential role of oxidative stress in osteoporosis is rapidly evolving, correcting the oxidative stress using an anti-oxidative agent might be a viable method to prevent osteoporosis. Vitamin E, a potent free radical scavenger, has been reported to possess bone-protecting properties through the alleviation of oxidative stress. In a longitudinal study of 405 Swedish men, urinary F_2_-isoprostane (a biomarker of oxidative stress) and serum αTF were measured at the age of 77 years whereas BMD was detected at the age of 82 years. In this study, Ostman et al. (2009) reported that elderly men with low serum levels of αTF had high oxidative stress and reduced BMD [102]. A double-blind, controlled clinical trial demonstrated a positive correlation between hip BMD and anti-oxidant activities (SOD and GPx) in elderly subjects. Besides, there was a diminution in the lipoperoxide level and less hip bone loss after the administration of ascorbic acid (1000 mg) and αTF (400 IU) [103]. Findings from an earlier in vivo study using 32-day-old chicks indicated that feeding the chicks with αTF (90 IU/kg diet) lowered the level of thiobarbituric acid-reactive substance (TBARS, an index for lipid peroxidation) in the plasma and liver of the animals, resulting in improved bone histomorphometric measurements in the trabecular bone [104]. Using a rat model of hindlimb unloading, αTF in a 15, 75, and 500 IU/kg diet dose-dependently improved oxidation parameters, evidenced by the increase in the ferric reducing ability of plasma (FRAP). Histological assessment also showed that αTF preserved the bone microstructure in the distal femur of animals [105]. In addition, αTF exerted beneficial effects on fracture healing. An animal study by Turk et al. (2004) found that advanced fracture healing and decreased levels of malondialdehyde (MDA) were detected in fractured male rats administered intraperitoneally with αTF (20 mg/kg) [106]. Another study using a rat model with osteoporotic fracture also suggested that 60 mg/kg αTF supplementation improved fracture healing by increasing the activities of anti-oxidant enzymes (SOD and GPx) in the bone of ovariectomized rats with fractures [107]. 

For T3, a recent randomised double-blinded placebo-controlled trial evaluated the effects of 12-week annatto T3 (10% γT3 and 90% δT3) supplementation on the levels of RANKL, OPG, bone markers (bone-specific ALP (BALP) and N-terminal telopeptide (NTX)) as well as oxidative stress biomarkers (8-hydroxy-2′-deoxyguanosine (8-OHdG)) in postmenopausal women with osteopenia (*n* = 87, aged 59.7 ± 6.8 years). There was a significant increase in the BALP/NTX ratio, but a decrease in the RANKL/OPG ratio and 8-OHdG concentrations in postmenopausal women receiving T3 supplementation relative to the placebo group [72]. In an in vitro study, Abd Manan et al. (2012) evaluated the effects γT3 on lipid peroxidation, anti-oxidant enzymes activities, and apoptosis of osteoblasts exposed to H_2_O_2_. Results indicated that γT3 at 1 μM prevented MDA elevation, reduced osteoblast apoptosis, and increased SOD, GPx, and CAT activities [41]. An in vivo study by Maniam et al. (2008) found that supplementation of 100 mg/kg palm-derived T3-rich fraction (14.62% αT3, 32.45% γT3, 23.93% δT3, and 18.43% αTF) reduced TBARS and increased GPx in the femur of normal adult male rats [108]. In contrast, these changes were not observed in the normal animals treated with αTF at a similar dose [108]. Results from a study by Nazrun et al. (2008) revealed that 60 mg/kg of palm T3 (30.7% αT3, 55.2% γT3, and 14.1% δT3) raised GPx and SOD activities as well as suppressing MDA levels in ovariectomized rats [109]. Comparatively, 60 mg/kg of αTF only raised SOD and the increment was not as high as with palm T3 [109]. 

Overall, both αTF and T3 exhibit beneficial effects on bone and fracture healing by maintaining a balanced profile between ROS production and levels of anti-oxidants. The current evidence also implies the anti-oxidative effects of T3 outweigh αTF in osteoporotic conditions. Several research gaps remain to be bridged by investigators. First, most of these studies focused on investigating the effects of vitamin E on SOD and GPx activities. The evaluation on the effects of vitamin E on CAT activity is indispensable since CAT and GPx work together to detoxify H_2_O_2_. Second, vitamin E potentially reactivates the anti-oxidant system to prevent skeletal impairment caused by oxidative stress. Therefore, the effects of vitamin E on the downstream targets of oxidative stress signalling, including the expression of FoxO, β-catenin, PPAR-γ, and NF-κB, requires further elucidation. 

### 3.4. Growth Factors

Growth factors are proteins that are usually stored in the extracellular matrix and are secreted for critical functions, such as cell division, matrix synthesis, and tissue differentiation, on the appropriate target cells [110,111]. They are also actively released after injury by the extracellular matrix, cells, and platelets [110]. Several growth factors have been proven to act on the skeleton [110]. The potential of vitamin E as a bone-protecting agent might be due to the ability of vitamin E to promote bone-related growth factors, including transforming growth factor-beta (TGF-β) [37,112], bone morphogenetic protein (BMP) [16,37,112,113], vascular endothelial growth factor (VEGF) [112], fibroblast growth factor (FGF) [112,114], and insulin-like growth factor-1 (IGF-1) [10].

The large superfamily of TGF-β consists of three mammalian isoforms of TGF-β (namely TGF-β1, TGF-β2, and TGF-β3) and BMP [115]. Both TGF-β and BMP are abundantly found in bone and cartilage, suggesting their crucial role in bone remodelling. Active TGF-β and BMP-2 bind to their respective tetrameric transmembrane serine/threonine kinase receptors, TGF-βR and BMPR, expressed on the surface of osteoblasts or osteoblast precursors to initiate intracellular signals via canonical suppressor of mothers against decapentaplegic-(SMAD-)dependent and non-canonical SMAD-independent pathways. In canonical SMAD-dependent signalling, the interactions of TGF-β/BMP-2 with their receptors cause phosphorylation of their immediate downstream targets (SMAD proteins), which then interact with SMAD4, forming SMAD complexes. Alternatively, TGF-β/BMP-2 activates the MAPK signalling cascade and the downstream target, activator protein-1 (AP-1), in non-canonical SMAD-independent signalling. The activated SMAD complexes and AP-1 proteins then translocate into the nucleus to direct the transcription of osteoblast specific genes [116].

Ahn et al. (2011) analysed gene expression during differentiation of human MSCs into osteoblasts after vitamin E stimulation. The expression levels of TGF-β1 and SMAD2 were upregulated >2-fold compared to the control [37]. However, in the study by Ibrahim et al. (2015), the authors reported that both the annatto-derived T3 alone group and the combined with lovastatin group showed increased expression of TGF-β2 and TGF-β3, but did not reach statistical significance when compared to the control group [112]. They concluded that TGF-β2 and TGF-β3 had maximal expressions during the early phase of fracture healing, which might be within one week after the fracture occurred [117]. According to Mundy (1996), TGF-β2 and TGF-β3 were expressed early to initiate signalling for BMP synthesis by osteoprogenitor cells, which is important during the early phase of fracture healing [118]. Thus, the TGF-β gene expressions may have been low when their levels were measured by the fourth week of fracture healing in the study. In another study, nicotine was intraperitoneally injected to the rats for two months to induce experimental osteoporosis and the expression of osteogenic genes was downregulated [113]. Palm vitamin E (60 mg/kg) significantly increased expression of BMP-2, OSX, and Runx-2 in these rats.

Annatto T3 was co-embedded with lovastatin (a hypolipidaemic agent) as particles for targeted delivery and then injected into muscle near the fractured tibia of ovariectomized rats. This combination was shown to upregulate BMP-2 expression. In this study, treatment with annatto T3 and lovastatin also increased expression of OCN and Runx-2 [112]. Another in-vivo study further supported the fact that co-supplementation of annatto T3 (60 mg/kg) and lovastatin for 2 months upregulated BMP-2 in ovariectomized rats. The improvement was greater in the group treated with the combination of T3 and lovastatin compared to the T3 alone group, thus proving that both agents might have additive or synergistic effects to improve the osteoporotic condition. It has been postulated that the upregulation of BMP-2 might be influenced by the suppression of the mevalonate pathway [16]. Both agents (T3 and lovastatin) suppress the mevalonate pathway by inhibiting isoprenoid intermediates, such as farnesyl pyrophosphate and geranylgeranyl pyrophosphate, to attach and form cytosolic prenylated proteins to small G proteins (Rac, Rho, Rap1, and Rab). The inhibited prenylation of RhoA, the first identified Rho member, has been reported to promote BMP-2-induced osteoblastic differentiation processes [119]. The prenylation process is also important for Rac and Rho proteins to mediate cytoskeletal changes, leading to membrane ruffling, lamellipodia formation, and stress fibres, eventually resulting in the activation of osteoclasts [120,121,122]. Thus, reduced protein prenylation may contribute to the disruption of the osteoclast cytoskeleton, inducing its apoptosis and reduced resorptive activity [123,124].

Interestingly, high intake of αTF and mixed T3/Tocomin (500 mg/kg diet) has no impact on bone mass, density, or turnover in rats during skeletal maturation. Although bone morphogenic protein receptor type 1B (BMPR1B) was found to be elevated in male rats, neither αTF nor mixed T3 had significant effects on other key genes related to osteoblast differentiation, including Runx-2, OCN, COL1α1, COL2α1, ALP, and osteopontin. More importantly, supplementation of αTF and mixed T3 for 18 weeks upregulated noggin (a BMP inhibitor) as well as downregulated SMAD5 and growth differentiation factor-10 (GDF-10, also known as BMP-3B) in the animals [114]. High levels of noggin expression in mature osteoblasts of 20-month-old C57BL/6J mice were often associated with impaired osteoblast formation, differentiation, and function [125]. These findings indicated that high doses of αTF or mixed T3 suppressed BMP signalling through the SMAD-dependent pathway, thus overcoming the other skeletal beneficial effects of vitamin E, and contributing to the negligible effect of high dietary αTF or mixed T3 during skeletal maturation [114].

VEGF-α, the most abundant VEGF isoform, is commonly used in studies investigating the biological effects of VEGF. Thus, VEGF-α is more commonly referred to as VEGF [126,127]. Deckers et al. (2000) demonstrated that VEGF expression was strongly increased during terminal osteoblast differentiation and reached maximum expression during the mineralization stage of bone repair [128]. VEGF has been reported to stimulate bone repair by promoting angiogenesis and bone turnover [129]. BMP-2 and VEGF-α are closely related in enhancing angiogenesis. It has been shown that BMP-2 stimulates the secretion of VEGF-α from osteoblasts [130]. Besides that, VEGF, which was given in combination with local sustained BMP-2 release, had significantly enhanced ectopic bone formation compared to BMP-2 alone [131]. Both the annatto T3 and combined (annatto T3 and lovastatin) treatments significantly increased the expression of VEGF-α (a crucial gene for fracture healing) in ovariectomized rats with fractures. The increase in VEGF expression was detected at week 4 after fracture induction to the tibiae of the rats [112]. 

The role of FGF in bone development, maintenance, and fracture healing has been established. FGF-2 is highly expressed in stromal cells and osteoblasts in bone [132]. FGF-2 exerts a differential function: The low molecular weight isoform of FGF-2 (LMWFGF-2) enhances bone formation whereas the high molecular weight isoform of FGF-2 (HMWFGF-2) suppresses bone mineralization [133]. Moreover, a study by Nakamura et al. (2005) reported that low doses of FGF-2 increased ectopic bone formation and high doses of FGF-2 inhibited bone formation in vivo [134]. LMWFGF-2 stimulates osteoblast differentiation and bone formation, which is in part mediated through modulation of the Wnt signalling pathway [135]. Loss of endogenous FGF-2 reduces the expression of Wnt ligand, low-density lipoprotein receptor-related protein 5 (LRP5) complex, β-catenin, and phosphorylation of glycogen synthase kinase-3 beta (GSK3β), which results in inhibition of the canonical Wnt/β-catenin signalling pathway [135]. On the other hand, HMWFGF-2 promoted the expression of SOST, a potent inhibitor for the Wnt/β-catenin signalling cascade, thus possessing inhibitory effects on bone mineralization. In short, the type of isoform and level of FGF-2 are the two important criteria for determining its net outcome on bone, whether positive or negative. A study by Tennant et al. (2017) demonstrated that administration of high dietary mixed T3 to 11-weeks male rats significantly increased the expression of FGF-2, however, the type of isoform was not mentioned [114].

With advancing age, the rate of bone formation might gradually decline. Age-related decline in IGF-1 levels was reported to be associated with bone loss in humans [136]. Bone cells respond to IGF-1 as it is ubiquitously found within bone and serum. The binding of IGF-1 to its tyrosine kinase receptor exerts anabolic effects on bone by transducing signals via the PI3K/protein kinase B (Akt) and MAPK signalling pathways. Upon IGF-1 receptor (IGFR) activation, downstream protein substrates are recruited, such as insulin receptor substrate 1 (IRS1) and Src homolog and collagen protein (SHC). IRS1 interacts with PI3K to phosphorylate phosphatidylinositol-4,5-diphosphate (PIP2) to phosphatidylinositol-3,4,5-trisphosaphate (PIP3). PIP3 further recruits phosphoinositide-dependent kinase 1 (PDK1) to phosphorylate Akt, causing the activation of Akt. Meanwhile, the MAPK network is activated through the formation of the SHC-Grb2-SOS complex. The role of PI3K/Akt and MAPK activation on skeletal acquisition has been established in vitro and in vivo [137,138]. Beyond the regulation of osteogenic differentiation factors through these pathways, IGF-1 secreted by osteoblasts in the bone tissue markedly induce directional migration (chemotaxis) of osteoblasts to the site of future bone formation [139]. In a study by Arjmandi et al. (2012), a diet containing high-doses of vitamin E (500 mg/kg diet) enhanced material and structural bone quality, which was accompanied by increased mRNA transcripts for OCN, COL1α1, and IGF-1 in old male mice [10].

Overall, BMP-2 is the most extensively studied growth factor by investigators for the effects of vitamin E on bone healing. Vitamin E potentially increased the levels of BMP-2 expression during the process of bone repair. Other growth factors, such as TGF-β, FGF-2, VEGF-α, and IGF-1, are also promoted by vitamin E during skeletal impairment, although there is a paucity of studies regarding these growth factors. Several limitations must be addressed by investigators. Firstly, a comparison between the two types of vitamin E (TF and T3) in bone healing is lacking. Some articles did not specify the type of vitamin E used in their experiments, making it difficult for a comparison to be made between the two subtypes of vitamin E. Secondly, growth factors other than BMP-2 warrant more studies to further investigate the mechanism of vitamin E as a bone-protecting agent.

### 3.5. Hormones

Parathyroid hormone (PTH), secreted by parathyroid glands, is a major hormone for the control of extracellular calcium and phosphate levels [140]. PTH increases serum calcium levels via its targets on the kidneys, intestines, and bone [141], thus suggesting its role in regulating bone remodelling. In the kidneys, PTH inhibits the reabsorption of phosphate, thus increasing the amount of ionized calcium in the blood. Besides, PTH stimulates the conversion of 25-hydroxyvitamin D (inactive form) into 1,25-dihydroxyvitamin D (active form). The production of activated vitamin D further enhances calcium uptake from the intestines [141]. PTH also breaks down the bone (the largest reservoir of calcium in the body) to release calcium. 

PTH exerts both catabolic and anabolic skeletal effects. Chronic hyperthyroidism is associated with bone loss whereby sustained PTH elevation stimulates excessive bone turnover [142]. The osteoclastogenic activity of PTH is mainly through its indirect action on osteoblasts due to the absence of functional PTH receptors on osteoclasts. PTH downregulates the expression of OPG and upregulates the expression of RANKL in osteoblastic cells via protein kinase A-(PKA)-induced activation of cAMP-response element-binding (CREB) protein [143,144]. PTH also stimulates the osteoblastic expression of MCP-1, which has a role in the recruitment, differentiation, and multinucleation of osteoclast precursors [145]. On the contrary, intermittent administration of PTH has been reported to increase bone mass [146]. The bone-building mechanism of PTH involves the activation of the canonical Wnt/β-catenin signalling pathway. An in vitro study by Tian et al. (2011) demonstrated that intermittent administration of PTH stimulated the gene expression of β-catenin, Runx-2, OCN, ALP, and bone sialoprotein (BSP) in osteoblastic MC3T3-E1 cells [147]. Another in vivo study also showed that the interaction between PTH and its receptors suppressed the synthesis of the osteocyte-derived Wnt antagonist, SOST, to further intensify the pro-osteoblastogenic signal [148].

The association between vitamin E, PTH levels, and bone health was previously investigated in an in vivo study. Norazlina et al. (2004) showed that Sprague-Dawley rats fed with a vitamin E-deficient diet had higher PTH levels as well as lower serum calcium levels and bone calcium contents in the lumbar bone as compared to control animals fed with standard rat chow [149]. However, vitamin E supplementation (αTF and αT3) at 60 mg/kg failed to reverse all these changes caused by a vitamin E deficiency. These findings reiterated the close relationship between vitamin E deficiency, secondary hyperparathyroidism, and vertebral bone loss even though replacing the vitamin E did not have any effects on PTH levels [149].

Leptin is initially a hormone predominantly produced by adipose tissue that acts on the hypothalamus and functions to regulate satiety and energy expenditure. The level of leptin is directly proportional to body fat content. Subsequently, leptin has emerged as a factor in the regulation of bone mass attributed to its diverse functions, including the promotion of haemopoietic and osteoblastic differentiation [150]. Adiponectin is another regulator that co-regulates energy homeostasis and bone metabolism. Similar to leptin, adiponectin is an adipocyte-derived hormone, but the level of adiponectin is inversely correlated with obesity. The presence of adiponectin receptors on osteoblasts suggests the role of adiponectin in bone homeostasis. Ample clinical evidence has indicated the relationship between these hormones (leptin and adiponectin) and BMD in osteoporotic patients of various causes [151,152,153].

Previous studies have disclosed the distinct effects of leptin on bone metabolism through central and peripheral pathways. Pioneering in vivo studies demonstrated that the activation of hypothalamic receptors of leptin in the brain suppressed osteoblastogenesis and increased osteoclast activity [154,155]. Two downstream molecular cascades involved are: (a) The downregulation of c-myc expression that increases production of cyclin D to inhibit osteoblast proliferation; and (b) the upregulation of RANKL via the PKA signalling pathway to promote resorption effects of osteoclasts [156]. Paradoxically, leptin binds to its receptors on bone marrow MSCs to enhance proliferation and differentiation of MSCs into an osteoblastic lineage via the direct peripheral pathway [157]. Leptin also binds with its receptor on osteoblasts to increase expression of OPG and inhibit secretion of RANKL [150]. Hence, the important question that remains unresolved for leptin signalling is the absolute dependency is on central or peripheral pathways as the nature of bone remodelling. On the other hand, adiponectin is known for its anti-atherogenic and insulin-sensitizing properties as well as its positive effects on bone development. In vitro studies showed that adiponectin promoted chondrocyte proliferation, proteoglycan synthesis, and matrix mineralization via an enhancement of the expression of type II collagen, Runx-2, BMP-2, and ALP activity [158,159]. The underlying mechanisms of adiponectin-mediated BMP-2 expression in osteoblastic cells involved the activation of the adenosine monophosphate-activated protein kinase (AMPK), p38 MAPK, and NF-κB pathways [158].

Since there is a link between these hormones and bone health, the evaluation of the effects of vitamin E (a bone-protecting agent) on the levels of leptin and adiponectin is necessary. Previous studies revealed that the rats fed with a high-carbohydrate high-fat diet experienced MetS, hyperleptinemia, and hypoadiponectinemia along with the impairment of trabecular microarchitecture as compared to normal control animals fed with standard chow diet. The intervention with palm T3 (21.9% αTF, 24.7% αT3, 4.5% βT3, 36.9% γT3, and 12.0% δT3) and annatto T3 (16% γT3 and 84% δT3) reduced the elevated leptin whereas only annatto T3 improved the level of adiponectin in the animals with bone loss due to MetS [31,32].

Based on the current state of knowledge, hormones seem to have a crucial role in both bone anabolism and catabolism. The underlying signalling of these hormones aforementioned in regulating bone metabolism is complex, thus meriting further investigation. In addition, the current available literature on the relationship between vitamin E and hormones using osteoporotic models is still lacking.

### 3.6. MicroRNA

MicroRNA (miRNA) is a small and highly conserved non-coding endogeneous RNA molecule containing 20–22 bases that regulates protein expression [160]. The role of miRNA has been widely established using in vitro studies. A panel of miRNAs targets transcription factor Runx-2 to control osteogenic and osteoblast differentiation [161]. Li et al. (2015) reported that the overexpression of miR-194 downregulated STAT1 and promoted nuclear translocation of Runx-2, leading to enhanced osteoblastogenesis [162]. In addition to the regulation of osteoblast differentiation, the expression of miRNA is also closely associated with osteoclast differentiation. Several miRNAs targeted the RANK/RANKL/OPG system to attenuate osteoclast formation and function. MiR-17/20a and miR-26a inhibited the RANKL expression [163,164]. Other miRNAs (e.g., miR-155) exhibited inhibitory effects on osteoclast differentiation via the TGF-β/SMAD signalling pathway in bone marrow-derived macrophages [165].

The role of vitamin E on miRNA expression has been investigated. Vitamin E deficiency resulted in a reduced concentration of miRNA-122a and miRNA-125b in the rat liver [166]. Similar outcomes were reported using Nile tilapia, whereby a diet deficient of DL-α-tocopherol acetate caused oxidative stress as well as downregulated miR-223, miR-146a, miR-16, and miR122 in the liver [167]. The evidence suggests that vitamin E potentially regulates the expression of miRNA, which might have a crucial role in the coordination of bone homeostasis. Thus, the role of vitamin E in modulating the expression of miRNA should be further evaluated in vivo using osteoporotic animal models and in vitro using various types of bone cells. 

## 4. Future Outlook and Conclusion

**Figure 1:**
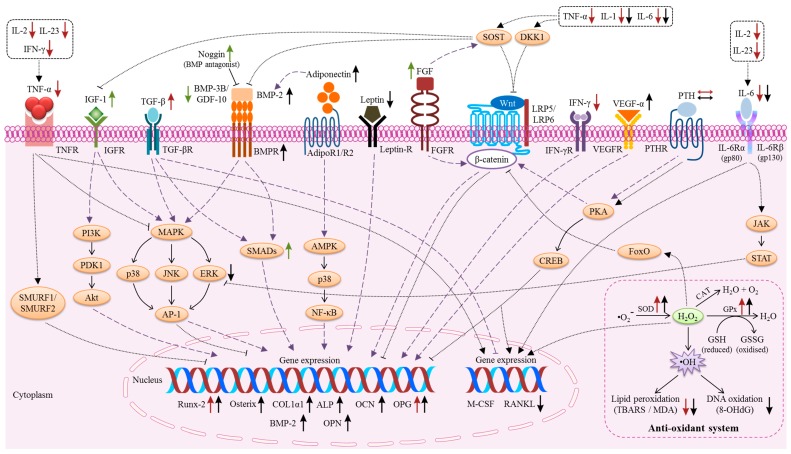
The signalling in osteoblasts for the regulation of bone metabolism. Under normal conditions, several essential signalling events can be activated to initiate the activity of bone formation (indicated by the purple dotted line, **— —**). Inflammation, oxidative stress, and hyperparathyroidism cause inhibition of bone formation via several pathways (indicated by the black dotted line, ······). The effects of TF (red arrows), T3 (black arrows) as well as high doses of TF and T3 (green arrows) are indicated.

**Figure 2:**
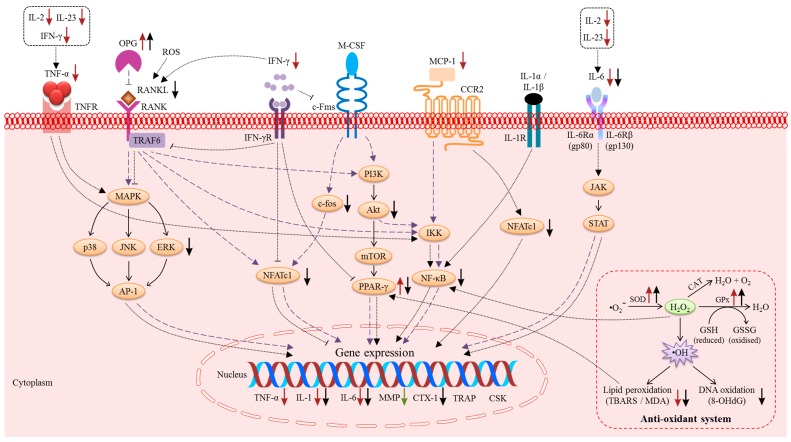
The signalling in osteoclasts for the regulation of bone metabolism. Under normal conditions, several essential signalling events can be activated to initiate the activity of bone resorption (indicated by the purple dotted line, **— —**). Inflammation and oxidative stress further activate bone resorption via several pathways (indicated by the black dotted line, ······). The effects of TF (red arrows), T3 (black arrows) as well as high doses of TF and T3 (green arrows) are indicated.

The underlying mechanism of the beneficial effects of vitamin E on bone is currently a topic of investigation. The endocrine and paracrine regulators direct the activation or inhibition of various downstream signalling pathways that contribute to the close coordination between bone resorption and formation. An increasing number of scientific studies have identified the molecular targets of vitamin E in regulating bone metabolism (Table 1). Two models summarizing the actions of vitamin E on osteoblasts (Figure 1) and osteoclasts are provided (Figure 2). Vitamin E modulated the levels of inflammatory mediators, ROS, growth factors, and hormones, which are locally and systemically released factors with a potent regulatory role in bone metabolism. Accordingly, vitamin E also plays a pivotal role in the modulation of RANK/RANKL/OPG, NF-κB, MAPK, and oxidative stress signalling.

The process of osteogenesis involves preosteoblast proliferation, osteoblast differentiation, and collageneous extracellular matrix formation [43]. Runx-2 is important for the differentiation of mesenchymal progenitor cells into osteoblasts whereas osterix has a role in both osteoblast differentiation and bone formation. Osterix is downstream of Runx-2 in osteoblast differentiation, this was supported by a study showing that Runx-2 was expressed in osterix-knockout mice, but osterix was not expressed in Runx-2 knockout mice [168]. Next, osterix regulates the downstream transcription of special AT-rich sequence-binding protein (Satb2) [169]. Satb2 functions to increase the expression of bone matrix proteins and osteogenic transcription factors during bone formation [170]. Firstly, the early osteoblast differentiation marker genes (such as COL1α1 and ALP) will be expressed. Subsequently, osteocalcin and BSP will be activated [43]. The current available evidence indicates that vitamin E targeted the Runx-2 mediated mechanism to increase the expression of bone formation-related genes.

Herein, we cautiously draw a conclusion that vitamin E has a role in orchestrating all these important regulators, which potentially favours a net increase in bone mass and ensures structural integrity of the skeleton.

Several research gaps require further exploration. Firstly, the reported positive outcomes of vitamin E on bone indicate the existence of anti-osteoporotic constituents in vitamin E. However, there is limited studies regarding the comparison of the anti-osteoporotic effects among the homologues of vitamin E. Secondly, multiple studies have highlighted the role of other potent regulators in bone remodelling. For instance, thyroid hormones, glucocorticoids, sex hormones (oestrogen and testosterone), and prostaglandins are the endocrine and paracrine regulators involved in bone formation and resorption. However, their role in formation and resorption activities associated with TF and T3 remain unknown. Thirdly, the effect of vitamin E on canonical Wnt/β-catenin signalling in bone cells (another key signalling pathway to achieve balanced formation and resorption) has not yet been elucidated and awaits further experimentation. New insights into the effects of vitamin E on these unexplored signalling on bone will open new prospects for developing novel therapies against osteoporosis. 

## Figures and Tables

**Table 1:** The targets for vitamin E in the regulation of bone metabolism.

Targets	References	Targets	References
Cytokines		Kinases	
IFN-γ ↓	[92]	Akt ↓	[171]
IL-1 ↓ ↓	[23,26,32,33,89,93]	ERK ↓	[71]
IL-2 ↓	[92]	Enzymes	
IL-6 ↓ ↓	[23,26,32,33,89,93]	GPx ↑ ↑	[107,108,109]
IL-23 ↓	[92]	SOD ↑ ↑	[107,109]
TNF-α ↓	[92]	Chemokines	
RANKL ↓	[15,19,70,72]	MCP-1 ↓	[92]
Growth factors		Hormones	
TGF-β1 ↑	[37]	Leptin ↓	[31,32]
FGF-2 ↑	[114]	Adiponectin ↑	[32]
VEGF-α ↑	[112]	Receptors	
GDF-10 / BMP-3B ↓	[114]	OPG ↑ ↑	[15,24]
IGF-1 ↑	[10]	BMPR1B ↑	[114]
Oxidative stress markers		Bone markers	
8-OHdG ↓	[72]	NTX ↓	[72]
TBARS/MDA ↓ ↓	[104,106,108,109]	ALP ↑	[43]
FRAP ↑	[105]	OCN ↑	[15,43]
Transcription factors		CTX-1 ↓	[15,17]
Osterix ↑	[15,43,113]	Genes	
Runx-2 ↑ ↑	[15,37,112,113]	BMP-2 ↑	[16,112,113]
PPAR-γ ↑ ↓	[19,160]	*ALPL* ↑	[19]
NFAT2 ↓	[70]	*SPP1* (osteopontin) ↑	[19]
NFATc1 ↓	[71]	Noggin ↑	[114]
NF-κB ↓	[71]	SMAD5 ↓	[114]
Protein		c-Fos ↓	[70,71]
COL1α1 ↑	[19,43]		

^1^ The effects of TF (red arrows), T3 (black arrows) as well as high doses of TF and T3 (green arrows) are indicated.

## Abbreviations

8-OHdG	8-hydroxy-2′-deoxyguanosine
Akt	Protein kinase B
ALP	Alkaline phosphatase
AMPK	Adenosine monophosphate-activated protein kinase
AP-1	Activator protein-1
BFR	Bone formation rate
BMD	Bone mineral density
BMP	Bone morphogenetic protein
BMPR1B	Bone morphogenic protein receptor type 1B
BSP	Bone sialoprotein
BV	Bone volume
CAT	Catalase
c-Fos	Fos proto-oncogene
c-Fms	Colony-stimulating factor-1 receptor
COL1α1	Collagen type 1 alpha 1
CREB	cAMP-response element-binding
CTX	Carboxyl-terminal telopeptide of type I collagen
DC-STAMP	Dendritic cell-specific transmembrane protein
DKK1	Dickkopf-related protein 1
DNA	Deoxyribonucleic acid
ERK	Extracellular signal-regulated kinases
ES	Eroded surface
FGF	Fibroblast growth factor
FoxO	Forkhead Box O
FRAP	Ferric reducing ability of plasma
GDF-10	Growth differentiation factor-10
GPx	Glutathione peroxidase
GSH	Reduced glutathione
GSK3β	Glycogen synthase kinase-3 beta
H_2_O	Water
H_2_O_2_	Hydrogen peroxide
HMWFGF-2	High molecular weight isoform of fibroblast growth factor-2
hs-CRP	High-sensitivity C-reactive protein
IFN	Interferon
IGF-1	Insulin-like growth factor-1
IGFR	Insulin-like growth factor-1 receptor
IL	Interleukin
IRS1	Insulin receptor substrate 1
JAK	Janus kinase
JNK	c-Jun N-terminal kinase
LMWFGF-2	Low molecular weight isoform of fibroblast growth factor-2
LRP5	Low-density lipoprotein receptor-related protein 5
MAPK	Mitogen-activated protein kinase
MCP-1	Monocyte chemoattractant protein-1
M-CSF	Macrophage-colony stimulating factor
MDA	Malondialdehyde
MetS	Metabolic syndrome
Micro-CT	Micro-computed tomography
mRNA	Messenger ribonucleic acid
MSCs	Mesenchymal stem cells
NFATc1	Nuclear factor of activated T-cells cytoplasmic 1
NF-κB	Nuclear factor-kappa B
NTX	N-terminal telopeptide
O_2_^•−^	Superoxide radical anion
O_2_	Oxygen
Ob.N	Osteoblast number
OCN	Osteocalcin
Oc.N	Osteoclast number
ODF	Osteoclast differentiation factor
OPG	Osteoprotegerin
OSX	Osterix
P1NP	Procollagen I intact N-terminal propeptide
PDK1	Phosphoinositide-dependent kinase 1
PI3K	Phosphatidylinositol-3-kinase
PIP2	Phosphatidylinositol-4,5-diphosphate
PIP3	Phosphatidylinositol-3,4,5-trisphosaphate
PKA	Protein kinase A
PPAR-γ	Peroxisome proliferator-activated receptor-gamma
PTH	Parathyroid hormone
PYD	Pyridinoline
Runx-2	Runt-related transcription factor-2
Satb2	Special AT-rich sequence-binding protein
SEM	Scanning electron microscope
SHC	Src homolog and collagen protein
SMAD	Suppressor of mothers against decapentaplegic
SMURF1	Suppressor of mothers against decapentaplegic ubiquitylation regulatory factor
SOD	Superoxide dismutase
SOST	Sclerostin
*SPP1*	Secreted phosphoprotein 1
sRANKL	Soluble receptor activator of nuclear factor kappa-B ligand
STAT	Signal transducers and activators of transcription
T3	Tocotrienol
TBARS	Thiobarbituric acid reactive substances
Tb.N	Trabecular number
Tb.Th	Trabecular thickness
Tcf	T cell factor
TF	Tocopherol
TGF-β	Transforming growth factor-beta
TNF-α	Tumour necrosis factor-alpha
TRAF6	Tumour necrosis factor receptor-associated factor 6
TRANCE	Tumour necrosis factor related activation-induced cytokine
RANK	Receptor activator of nuclear factor kappa-B
RANKL	Receptor activator of nuclear factor kappa-B ligand
ROS	Reactive oxygen species
VEGF	Vascular endothelial growth factor
Wnt	Wingless-related integration site
